# A xenograft model for venous malformation

**DOI:** 10.1007/s10456-018-9624-7

**Published:** 2018-05-21

**Authors:** Jillian Goines, Xian Li, Yuqi Cai, Paula Mobberley-Schuman, Megan Metcalf, Steven J. Fishman, Denise M. Adams, Adrienne M. Hammill, Elisa Boscolo

**Affiliations:** 1 Division of Experimental Hematology and Cancer Biology, Cancer and Blood Diseases Institute, Cincinnati Children’s Hospital Medical Center, Cincinnati, OH 45229-3039 USA; 2 Division of Hematology, Cancer and Blood Diseases Institute, Cincinnati Children’s Hospital Medical Center, Cincinnati, OH USA; 3 Departments of Surgery, Harvard Medical School, Boston Children’s Hospital, Vascular Anomalies Center, Boston, MA USA; 4 Department of Hematology, Harvard Medical School, Boston Children’s Hospital, Vascular Anomalies Center, Boston, MA USA; 5 Department of Pediatrics, University of Cincinnati College of Medicine, Cincinnati, OH USA

**Keywords:** Vascular anomaly, Venous malformation, TIE2, PI3K, AKT, Endothelial cell, Vascular, Patient-derived xenograft, Rapamycin, Sirolimus

## Abstract

Vascular malformations are defects caused by the abnormal growth of the vasculature. Among them, venous malformation (VM) is an anomaly characterized by slow-flow vascular lesions with abnormally shaped veins, typically in sponge-like configuration. VMs can expand over years causing disfigurement, obstruction of vital structures, thrombosis, bleeding, and pain. Treatments have been very limited and primarily based on supportive care, compression garments, sclerotherapy, and/or surgical resection. Sirolimus treatment has recently shown efficacy in some patients with complicated vascular anomalies, including VMs. Activating somatic TIE2 gene mutations have been identified in up to 60% of VMs and *PIK3CA* mutations have been found in another 25%. Here, we report a xenograft model of VM that reflects the patients’ mutation heterogeneity. First, we established a protocol to isolate and expand in culture endothelial cells (VM–EC) from VM tissue or VM blood of nine patients. In these cells, we identified somatic mutations of *TIE2, PIK3CA*, or a combination of both. Both *TIE2* and *PIK3CA* mutations induced constitutive AKT activation, while *TIE2* mutations also showed high MAPK–ERK signaling. Finally, VM–EC implanted into immune-deficient mice generated lesions with ectatic blood-filled channels with scarce smooth muscle cell coverage, similar to patients’ VM. This VM xenograft model could be instrumental to test the therapeutic efficacy of Sirolimus in the presence of the different *TIE2* or *PIK3CA* mutations or to test for efficacy of additional compounds in targeting the specific mutated protein(s), thus enabling development of personalized treatment options for VM patients.

**Electronic supplementary material:**

The online version of this article (10.1007/s10456-018-9624-7) contains supplementary material, which is available to authorized users.

## Introduction

Venous malformation (VM) is a congenital chronic condition that can be severely disfiguring [1, 2]. The incidence of VM is approximately 1 in 5–10,000 [2, 3]. VMs consist of endothelial-lined dilated slow-flow dysmorphic venous channels, typically in a sponge-like configuration with impaired smooth muscle cell coverage. VMs can cause significant pain, obstruction of organ function, and in some cases, localized intravascular coagulopathy resulting in risk of bleeding and thrombosis. Current treatment of VM is based on ablation of the abnormal vessels by sclerotherapy or removal by surgical excision; however, these approaches are not curative and regrowth is common [2]. In recent clinical trials, treatment with the mTOR inhibitor Sirolimus has shown efficacy in some patients affected by VMs [4, 5].

Activating germline or somatic *TEK* (*TIE2*) mutations are associated with VM [6, 7]. TIE2 is an endothelium-specific receptor tyrosine kinase that regulates both maintenance of vascular quiescence and promotion of angiogenesis. More recently, somatic mutations in the catalytic subunit of class I phosphoinositide 3-kinases (*PIK3CA)*, reported in several types of cancer [8], overgrowth syndromes [9–11], and lymphatic malformations [12–14], have been identified in about 20–25% of VM cases [15–17].

Murine models for VM have been reported. The first model is based on subcutaneous injection of human umbilical vein endothelial cells (HUVEC) engineered to express the most frequent mutation identified in VM patients *TIE2* p.L914F [5], or other *TIE2* mutations [18]. The second system relies on the transgenic expression of *PIK3CA* p.H1047R in Sprr2f^+^ cells (epithelial and endothelial), in the embryonic mesoderm or in VE-Cadherin^+^ cells [15, 16, 19].

Here, we isolated and characterized EC from tissue or lesional blood from VM patients (VM–EC) and determined the presence of *TIE2* (p.L914F), *PIK3CA* (p.H1047R, C420R), or combination of both (*TIE2* p.R915C and *PIK3CA* p.Q546K) somatic mutations. We determined that the *TIE2* mutation was not present in the non-endothelial cells obtained from VM samples. Furthermore, we established a xenograft model of VM by subcutaneous injection of the VM–EC. The mutated VM–EC formed enlarged blood-filled vessels with scarce smooth muscle cell coverage, akin to human VM. This model is reflective of the range of mutations found in patients.

## Results

### Isolation and characterization of endothelial cells from tissue and blood derived from VM lesions

**Fig. 1:**
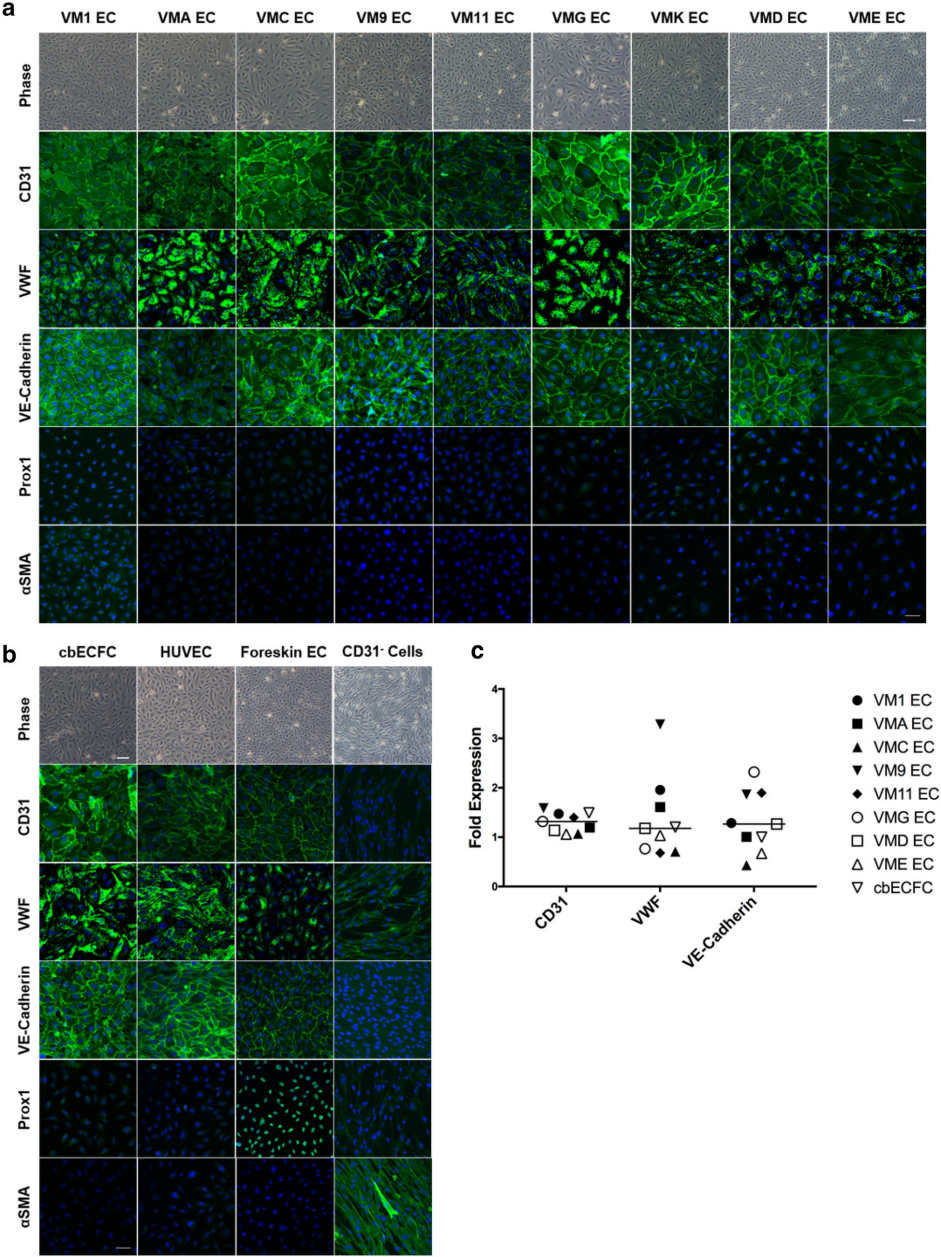
Characterization of VM–EC morphology and endothelial marker expression. **a** VM–EC at 80–90% confluency stained positive for endothelial markers CD31, vWF, VE-Cadherin, and negative for lymphatic marker Prox1 and smooth muscle marker αSMA, similar to **b** control EC (cord blood endothelial colony forming cells, cbEFCF; human umbilical vein endothelial cells, HUVEC; Foreskin EC). Foreskin EC were a positive control for Prox1 staining, and CD31^−^ cells (non-EC isolated from VM1 tissue) were a positive control for αSMA. Specific markers (green), nuclei (blue). Scale bars: phase 100 µm; immunofluorescence 50 µm. **c** qRT-PCR of CD31, VWF, and VE-Cadherin gene expression of VM–EC and cbECFC, normalized to HUVEC. *n* = 3 independent repeats, in triplicate

EC were successfully isolated from 9 VM patients (3 solid tissues and 6 lesion blood samples collected during sclerotherapy procedure) (Table 1). VM–EC monolayers presented with a homogeneous cobblestone appearance up to passage 7 (Supplemental Fig.S1) and expressed EC-specific markers CD31, vonWillebrand Factor (vWF), and vascular endothelial (VE)-Cadherin (Fig. 1a), similarly to normal, control EC (Fig. 1b). VM–EC did not show expression of lymphatic marker Prox1 nor smooth muscle alpha actin (αSMA) (Fig. 1a). Quantitative real-time polymerase chain reaction (qRT-PCR) revealed that each VM–EC population expressed EC-specific genes at similar levels (*p* > 0.12) compared to human umbilical vein endothelial cells (HUVEC) and cord blood endothelial colony forming cells (cbECFC) (Fig. 1c).

**Table 1:** Patients’ VM samples

Patient ID	VM location	Age at collection (years)	Sex	Endothelial cell source	Date obtained	PIK3CA mutation	TIE2 mutation	Presence in blood or saliva	Mutant allelic frequency in VM–EC
1	Leg	15	F	Tissue	1/7/14		L914F	n/a	128/273 (46.9%)
A	Thigh	4	F	Lesion Blood	5/8/15		L914F	n/a	n/a
C	Hip, pelvis, perineum, leg	15	F	Lesion Blood	8/25/15		L914F	No	n/a
D	Lower extremity IM	18	F	Lesion Blood	10/13/15	H1047R		No	n/a
E	Calf IM	18	F	Lesion Blood	11/10/15	C420R		No	34/57 (59.6%)
9	Buttock, knee, leg	9	M	Tissue	8/22/14		L914F	n/a	n/a
11	Arm	3	M	Tissue	12/22/16		L914F	n/a	n/a
G	Chest	20	F	Lesion Blood	4/13/16		L914F	No	n/a
K	Thigh, calf, ankle	12	F	Lesion Blood	3/4/17	Q546K	R915C	No	n/a

*IM* intramuscular, *n/a* not available; no mutation not detected

### *TIE2* and *PIK3CA* somatic mutations exist in VM–EC

**Fig. 2:**
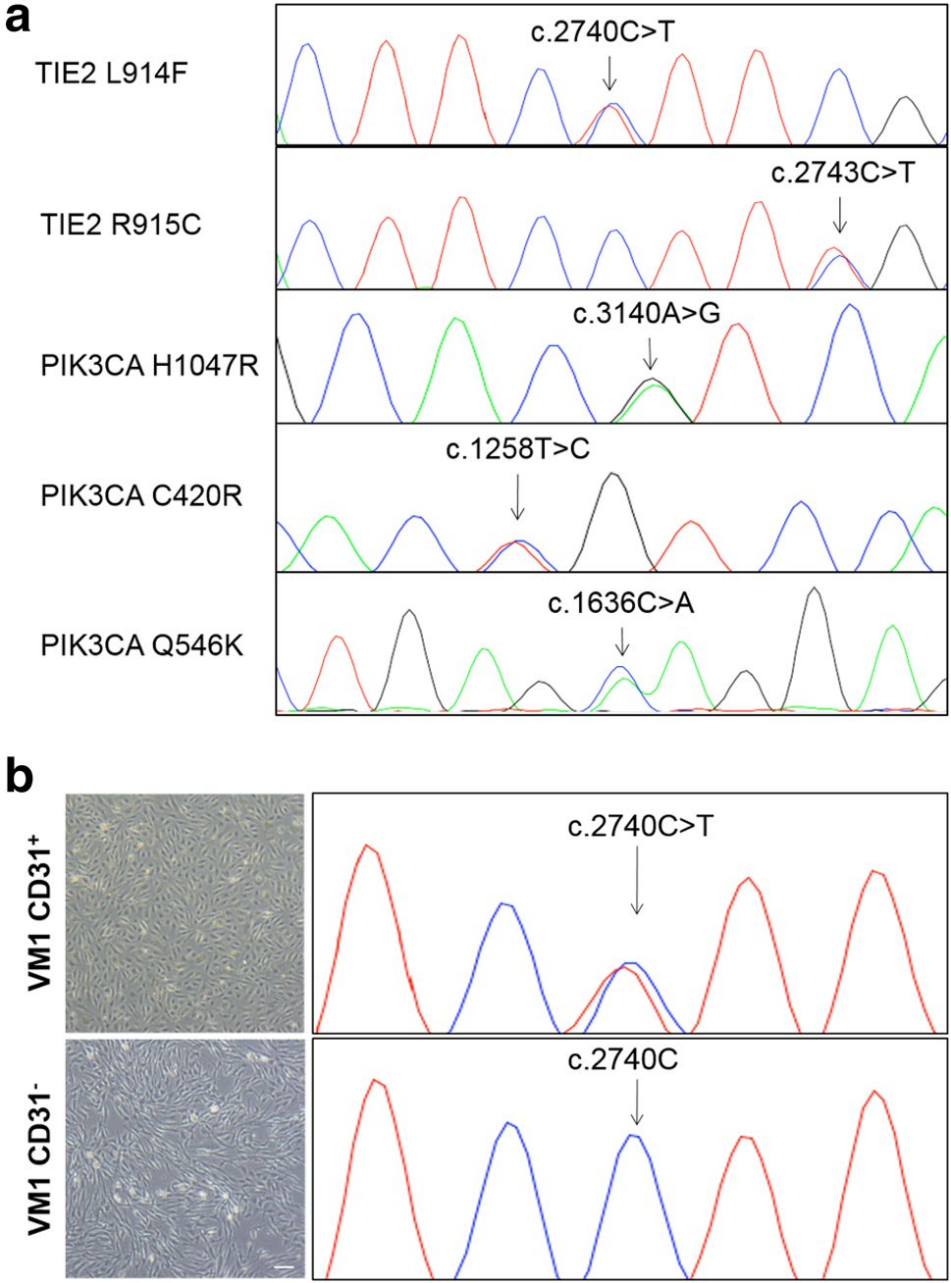
Sequencing of VM–EC (CD31^+^) and non-EC (CD31^−^) for *TIE2* and *PIK3CA* mutations. **a** DNA Sanger sequencing of VM–EC showed the presence of a double peak at *TIE2* c.2740C > T (p.L914F) (VM1 EC), c.2743C > T (p.R915C) (VMK EC); *PIK3CA* c.3140A > G (p.H1047R) (VMD EC), c.1258T > C (p.C420R) (VME EC), c.1636C > A (p.Q546K) (VMK EC); **b** EC (CD31^+^) and non-EC (CD31^−^) from same VM patient (VM1) were sequenced to compare the presence of somatic mutation (*TIE2* c.2740C > T, p.L914F); *n* = 5, see Table 2. Scale bar: 100 µm

VM–EC DNA Sanger sequencing analysis was performed for *TIE2* exon 17 (tyrosine kinase domain). If initial analysis did not detect a p.L914F mutation, we carried out *TIE2* next-generation sequencing (NGS) to screen for other *TIE2* mutations. Next, primers amplifying *PIK3CA* exons 7, 9 (α-helical domain), and 20 (tyrosine kinase domain) were used to further determine, by DNA Sanger sequencing, the presence of mutations frequently associated with vascular anomalies (at sites p.C420, E542, E545, and H1047) [9, 11, 15–17]. *TIE2* p.L914F mutations were identified in 6/9 VM–EC, making this the most frequent mutation in our study and in agreement with previous literature [6, 20]. Mutually exclusive *PIK3CA* mutations were present in 2/9 VM–EC (p.H1047R and C420R). Interestingly, sequencing analysis revealed a simultaneous expression of *TIE2* p.R915C and *PIK3CA* p.Q546K mutations in 1/9 VM–EC (VMK EC) (Table 1; Fig. 2a). 7/7 single cell-derived clonal populations expressed both the *TIE2* and the *PIK3CA* mutations, suggesting VMK EC is a pure population of double-mutant EC (Supplemental Fig. S2). Mutated allele frequencies in VM–EC that were subjected to *TIE2* gene or whole exome NGS equaled 46.9% in VM1 EC (*TIE2* c.2740C > T) and 59.6% in VME EC (*PIK3CA* c.1258T > C) (Table 1), suggesting the VM–EC populations tested contain predominantly heterozygous mutant cells. The *TIE2* or *PIK3CA* mutation(s) were also analyzed in five available CD31 negative (CD31^−^, non-endothelial) cell populations obtained from the same patient sample. No *TIE2* p.L914F variant was detected in these CD31^−^ cell populations (Table 2; Fig. 2b). Mutations found in VM–EC were further confirmed to be somatic by Sanger sequencing of peripheral blood or saliva genomic DNA from the same patient (Table 1).

**Table 2:** Mutation analysis in CD31^+^ and CD31^−^ cells from VM patients

Patient ID	*TIE2*/ *PIK3CA* mutation detected	
	Endothelial cells (CD31^+^)	Non-endothelial cells (CD31^−^)
1	*TIE2* L914F	No
A	*TIE2* L914F	No
9	*TIE2* L914F	No
11	*TIE2* L914F	No
G	*TIE2* L914F	No

No: Not detected

### Patient-derived VM–EC xenograft model recapitulates formation of enlarged blood vessels as seen in patients’ VM lesions

**Fig. 3:**
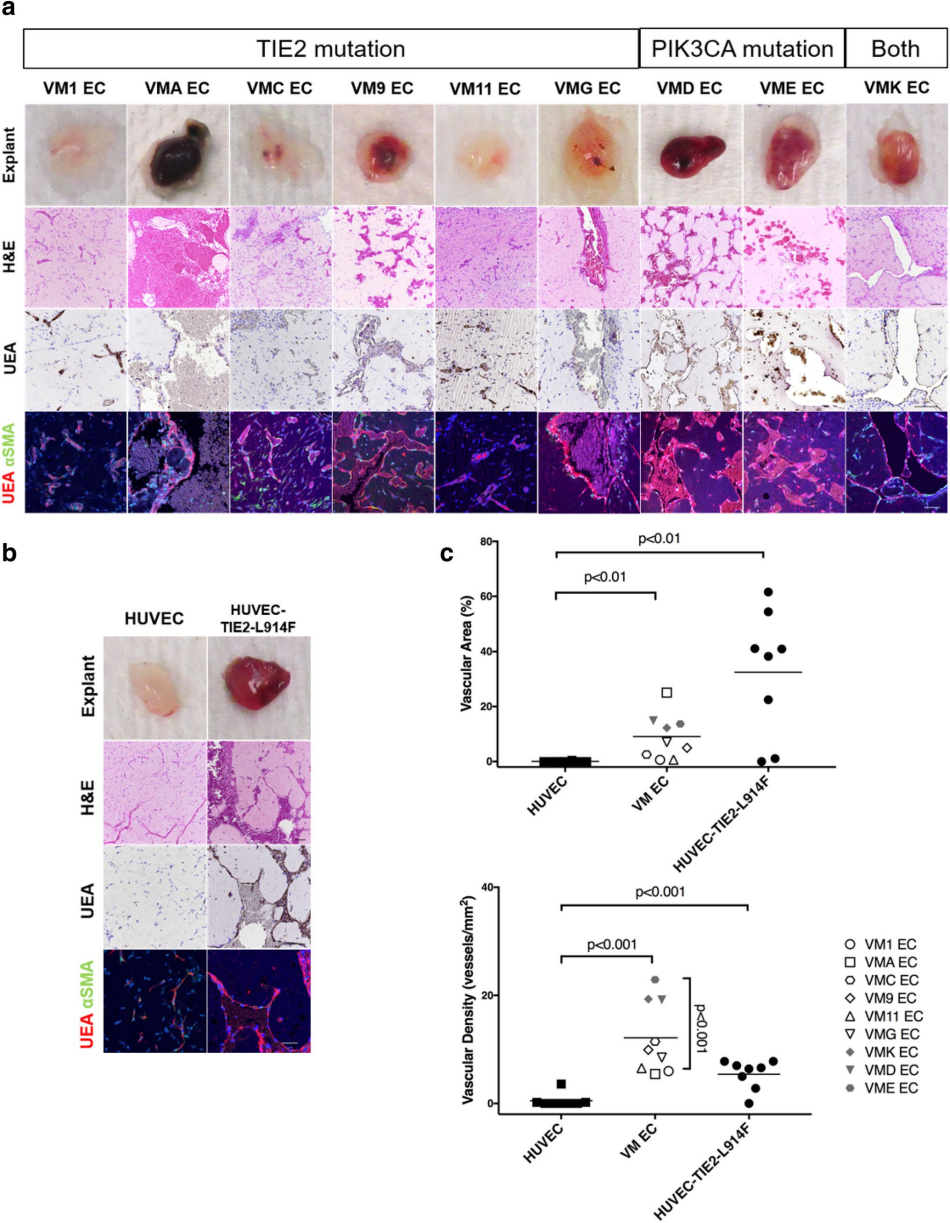
Xenograft model of VM, lesion explants from injected VM–EC. **a** VM–EC, **b** HUVEC, and HUVEC–TIE2–L914F cells were injected subcutaneously on both backsides of immune-deficient mice. Lesion explant photo (top), staining of hematoxylin and eosin (H&E) (second row), immunohistochemistry (IHC) of Ulex europaeus I (UEA) (third row), and immunofluorescence (IF) of UEA (red) and αSMA (green), nuclei (blue) (bottom); Scale bars: H&E, IHC 100 µm; IF 50 µm. **c** Vascular area quantification as %, and vessel density as vessels/mm^2^, 5 fields/lesion. *n* = 7–8 mice with 2 lesions each, per group

VM–EC were expanded in culture and injected subcutaneously into immune-deficient mice to determine if they could recapitulate the histological features seen in VM patient tissue. Explanted lesions were visibly vascularized at day 9 (Fig. 3a). Hematoxylin and eosin (H&E) staining of lesion sections showed numerous enlarged, blood-filled vessels similar to patient VM histology (Supplemental Fig. S3) and a VM murine model based on HUVEC–TIE2–L914F [5] (Fig. 3b). Normal HUVEC and HUVEC–TIE2–WT (wild type) injected explants did not show evidence of vascularization (Fig. 3b and Supplemental Fig. S4). VM–EC and HUVEC–TIE2–L914F explant sections showed positive staining for human-specific EC-marker Ulex europaeus agglutinin I (UEA), revealing that the lining of vascular channels consisted predominantly of the injected human-EC rather than invading mouse-EC (Fig. 3a). Isolectin B4 (IB4) staining further confirmed that enlarged blood vessels in the VM–EC explants were not mouse EC-derived (Supplemental Fig. S5, and positive/negative controls in Supplemental Fig. S6). Staining with αSMA showed scarce smooth muscle cell coverage (Fig. 3a), as previously reported in VM patient tissue [7]. Quantitative analysis of VM–EC explants at day 9 revealed a significant increase in vascular area (*p* < 0.01) and vascular density (*p* < 0.001) when compared to normal HUVEC explants (Fig. 3c). Furthermore, *PIK3CA* mutated VM–EC generated VM lesions with a significantly higher (*p* < 0.001) vascular density (Fig. 3c).

### Constitutive AKT activation downstream of the *TIE2* and *PIK3CA* mutations in VM–EC

**Fig. 4:**
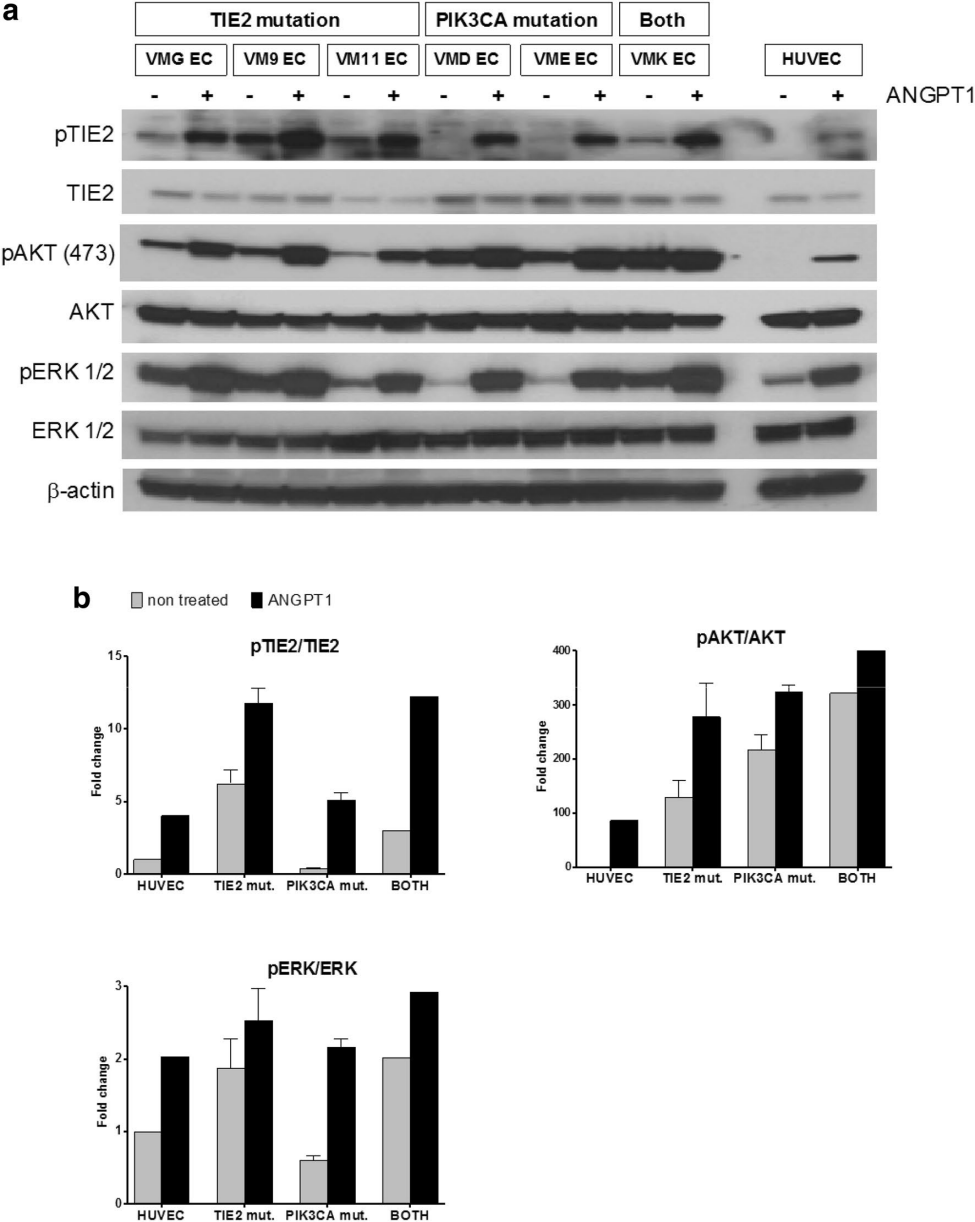
PI3K–AKT and MAPK–ERK signaling pathway activation in VM–EC. **a** Western blot analysis of TIE2, AKT, and ERK activation levels in VM–EC and HUVEC non-treated or stimulated for 15 min with Angiopoietin 1 (ANGPT1) 1 µg/ml. β-actin served as loading control. **b** Densitometric analysis of phospho-TIE2, phospho-AKT473, and phospho-ERK western blot bands relative to total TIE2, AKT, and ERK, respectively. Data are normalized to HUVEC non-treated

To analyze the activation status of the TIE2–PI3KCA downstream pathways in the VM–EC with different types of mutation, we performed immunoblot analysis of phospho-TIE2, phospho-AKT, and phospho-ERK levels. VM–EC (G,9,11,K) expressing *TIE2* mutations (p.L914F and p.R915C) showed constitutive activation of the TIE2 receptor that was not present in the *PIK3CA* mutated VM–EC (D, E) and in HUVEC (Fig. 4a). AKT activation was elevated downstream of both *TIE2* (p.L914F) and *PIK3CA* mutations (p.H1047R, p.C420R) and was higher in VM–EC expressing *PIK3CA* variants. Conversely, ERK activation was higher in the *TIE2* p.L914F VM–EC when compared to VM–EC expressing *PIK3CA* mutations (Fig. 4b). VM–EC and HUVEC responded to ANGPT1 stimulation by activating both the PI3K–AKT and the MAPK–ERK pathways. VMK EC expressing both *TIE2* and *PIK3CA* mutations (double *TIE2* p.R915C/*PIK3CA* p.Q546K) showed higher phospho-AKT levels when compared to both *TIE2* and *PIK3CA* single mutation VM–EC and phospho-ERK levels were similar to the *TIE2* p.L914F.

## Discussion

**Fig. 5:**
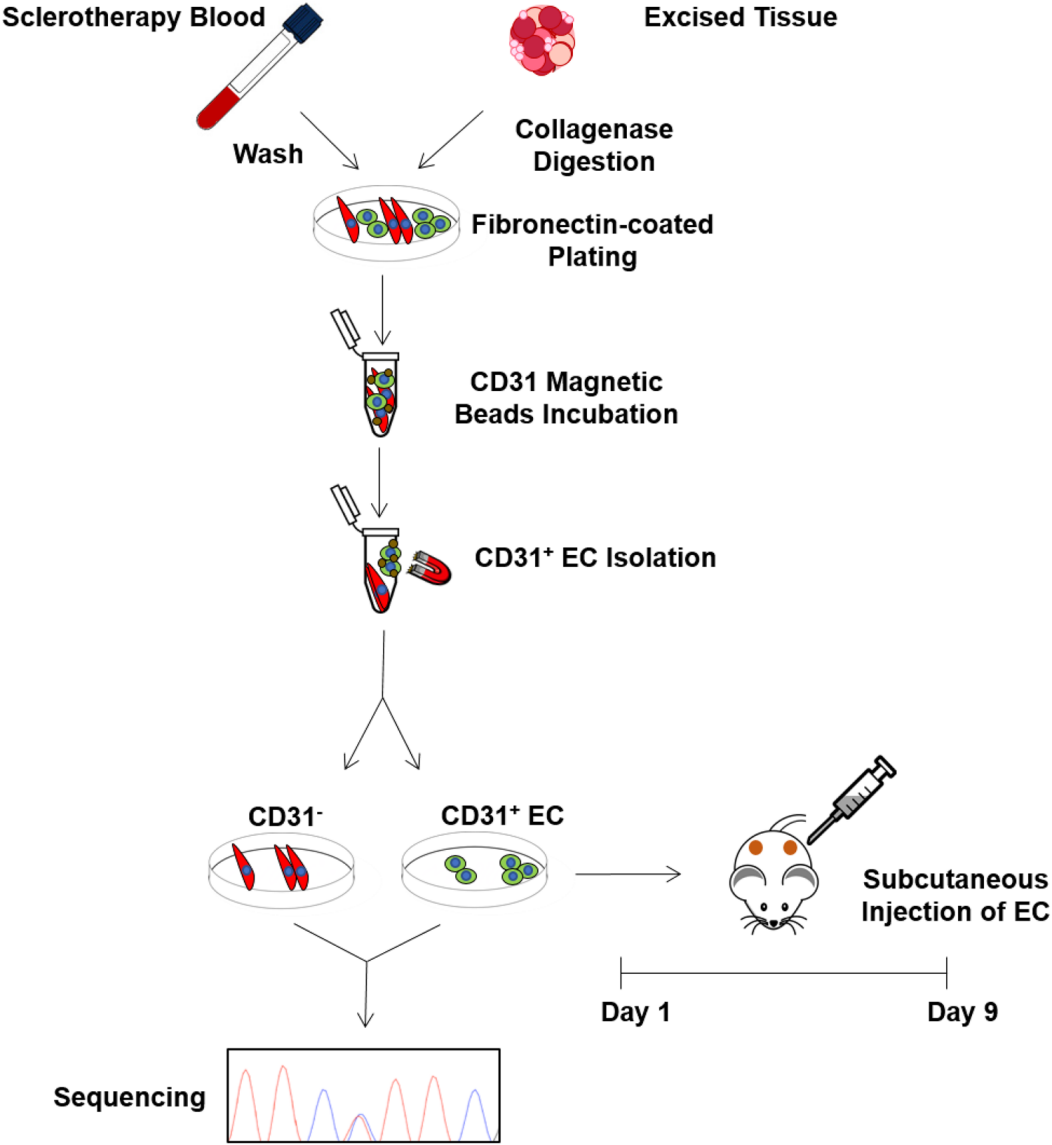
Schematic of VM–EC xenograft model generation. Endothelial cells isolated from patient VM lesion solid tissue or blood are plated and, when 80% confluency is reached, are selected by CD31-conjugated immunomagnetic beads and expanded. CD31^+^ cells (VM–EC) are injected subcutaneously into an immune-deficient mouse to generate a xenograft model of VM. Both CD31^+^ and CD31^−^ cells are subjected to Sanger sequencing to confirm the presence of somatic *TIE2* or/and *PIK3CA* mutation

Here, we show successful isolation and propagation of EC from both lesional blood and solid tissue types from VM patient samples. We have also determined that *TIE2* mutations are specifically expressed in the EC population of the VM tissue. We generated a novel xenograft model by injecting mutated EC derived from VM patients (Fig. 5). This murine model recapitulates the patients’ VM histology and can be a very useful tool to further study the pathology of VM on a patient-to-patient basis.

The feasibility of EC isolation from VM was first demonstrated with the use of solid VM lesion tissue, including intramuscular VM samples [21, 22]. In our study, in addition to solid tissue samples, we established a protocol to isolate EC from the blood aspirated from VM lesions during sclerotherapy procedure. This fluid is normally discarded and is more frequently available than solid tissue resected by surgery, as sclerotherapy is a less invasive procedure and commonly used to treat VM patients [2, 23]. Furthermore, we have been able to detect somatic *TIE2* and/or *PIK3CA* mutations in the EC derived from VM blood, suggesting this as a potential strategy to identify somatic mutations in patients that do not undergo surgery or solid tissue biopsy.

Somatic *TIE2* mutations are detected in about 34–61.5% of VM patients and they are mostly located on the tyrosine kinase domain of the TIE2 receptor [15–17, 20]. In our study, we predominantly identified the *TIE2* variant p.L914F (6/9), which is reported to be the most frequent among the *TIE2* mutation types [6, 20].

Activating *PIK3CA* mutations are associated with about 20–25% of sporadic VM cases and are reported as mutually exclusive with *TIE2* mutations [15–17]. Here, we show that 1/9 VM–EC expressed both a *TIE2* p.R915C and a *PIK3CA* p.Q546K mutation and that both mutations are present in each cell within our VMK EC population. *PIK3CA* mutation at p.Q546 site has been previously reported as single mutation and the *TIE2* mutation p.R915C as mosaic single or double *TIE2* mutation in combination with p.Y897H/C [17, 18]. While these studies investigated the presence of the somatic *TIE2* or *PIK3CA* mutation(s) in VM tissue, here we performed in-depth studies to show that the *TIE2* mutation p.L914F is exclusively expressed by the EC population within the lesion. We speculate that this is true for every *TIE2* and *PIK3CA* mutation, although we cannot confirm as we were not able to grow CD31 negative cells from all specimens obtained.

We have established that both *TIE2* and *PIK3CA* mutations result in constitutive AKT activation in VM–EC, similarly to HUVEC overexpressing *TIE2* p.L914F or *PIK3CA* p.H1047R [17]. Furthermore, our results determined that *PIK3CA* mutations are stronger AKT activators while *TIE2* mutations also affect the MAPK–ERK signaling.

We have previously reported a *TIE2* mutation-dependent murine model of VM based on subcutaneous injection of HUVEC–TIE2–L914F. These mice form VM lesions where the injected cells remodel into massively enlarged blood vessels that expand over time [5]. More recently, some studies reported animal models of VM consisting of transgenic mice that express *PIK3CA* p.H1047R in the vasculature [15, 16, 19]. These models have been instrumental to demonstrate the efficacy of mTOR inhibition in preventing VM lesion growth, but fail to reflect the heterogeneity of the mutations identified within VMs and the patients’ genetic background. In vitro, rapamycin treatment was shown to inhibit AKT activation in HUVEC expressing *TIE2* p.L914F, p.R1099X or *PIK3CA* p.E542K, p.E545K, and p.H1047R [5, 17]. Additional in vivo studies are needed to confirm drug efficacy on the different mutation types. Here, we sought to use patient-derived VM–EC to generate a xenograft model that would be more representative of the VM population and that could be used as a platform for future testing of drug efficacy on VM lesions with a range of *TIE2* and/or *PIK3CA* variants. All of the VM–EC we analyzed formed aberrant vascular networks when injected in vivo. In addition, we identified an association between the high levels of AKT activation in PIK3CA mutated VM–EC and vascular density in the xenograft. Furthermore, we strongly encourage future studies with a larger sample size to investigate association between number and/or diameter of the VM blood vessels and the mutation type.

## Materials and methods

### Tissue samples

Patient tissue samples were obtained from participants after informed consent from the Collection and Repository of Tissue Samples and Data from Patients with Tumors and Vascular Anomalies (IRB #2008-2001 per institutional policies) at Cincinnati Children’s Hospital Medical Center (CCHMC), Cancer and Blood Disease Institute (CBDI), and with approval of the Committee on Clinical Investigation at Boston Children’s Hospital. Samples include excised tissue, blood/fluid aspirated at the time of sclerotherapy, peripheral blood, and buccal mucosal swabs. Collected data and identifying names were stored in a secure database maintained by the CBDI. A unique patient number, created by the database, was assigned to each sample. This was further de-identified by creating a patient ID for use in this study.

### Cell culture

Solid tissue samples were minced and digested in 5 ml of Dulbecco’s modified Eagle medium (DMEM) (Gibco) supplemented with 2% fetal bovine serum (FBS) (Hyclone), 1X Ca^2+^/Mg^2+^, and 1 mg/ml of collagenase A (Roche) dissolved in phosphate-buffered saline solution (PBS) (Thermofisher) at 37 °C for 30 min. The digested tissue was homogenized with a pestle with 5 ml of PBS supplemented with 0.5% bovine serum albumin (BSA) (Sigma) and 2% penicillin–streptomycin–glutamine (PSG) (Corning) four times. Homogenized tissue was then filtered through a 100 µm cell strainer to remove fragments, followed by centrifugation for 5 min at 1500 rpm. Lesional blood was diluted in PBS to a final volume of 40 ml and centrifuged at 1000 rpm for 5 min. Tissue culture plates, 100 mm, were coated with 1 µg/cm^2^ of fibronectin (Millipore) in 0.1 M Na_2_CO_3_, pH 9.4, coating buffer, incubated at 37 °C, 5% CO_2_ for 20 min, and washed with PBS. The pellet from both tissue or fluid samples was re-suspended in Endothelial Growth Medium-2 (EGM-2) (Lonza), with 20% FBS, 1% PSG, and seeded onto fibronectin-coated plates. Colonies of endothelial cells appeared evident within 2–3 weeks from cell seeding. When cells reached 80% confluency, they were trypsinized and pelleted by centrifugation for 5 min at 1500 rpm and EC were purified with anti-CD31 antibody-conjugated magnetic beads (Dynal) following the manufacturer’s instructions. Retrovirally transfected HUVEC–TIE2–WT or TIE2–L914F were obtained as previously described [5, 24], in brief: full-length TIE2–WT or TIE2–L914F were cloned into pMXs vector and packaging cell line 293-GPG VSV-G was transfected for retrovirus production with Fugene 6 (Roche). All cell types were cultured in identical conditions: EGM-2 20% FBS, 1% PSG, on fibronectin-coated plates at 37 °C, 5% CO_2_.

### Immunocytochemistry

Photos of cell monolayers, passage 3–7, were taken with a phase-contrast microscope (Zeiss) using ZenLite Software. Immunocytochemistry was performed when cells reached 80–90% confluency. Cells were fixed with cold methanol (Fisher Chemicals) at 4 °C for 10 min and blocked in 5% horse serum (Vector Laboratories) in PBS. Primary antibody incubation with anti-CD31 (1:50, Dako), vonWillebrand Factor (vWF) (1:100, Dako), αSMA (1:500, Sigma), VE-Cadherin (1:50, Santa Cruz), and PROX1 (1:50, R&D Systems) was performed for 1 h at room temperature (RT). Fluorescein isothiocyanate (FITC)-conjugated secondary antibodies (1:200, Vector Laboratories) were used for 1 h at RT. Samples were mounted using Prolong Gold with 4′,6-diamidino-2-phenylindole (DAPI) (Life Technologies) and images acquired using C2 confocal microscope (Nikon).

### cDNA synthesis and quantitative RT-PCR

VM–EC, cbECFC, and HUVEC monolayers at passage 3 or 4 were lysed and homogenized using Qiashredder (Qiagen) and RNA isolated with RNeasy Minikit (Qiagen). RNA concentration and quality were determined with Nanodrop 2000c Spectrophotometer (Thermofisher). Synthesis of cDNA was performed using 1.0 µg of RNA and iScript cDNA Synthesis Kit (Biorad). Quantitative real-time PCR (qRT-PCR) was performed using 10 ng of cDNA in 20 µl reaction using SsoAdvanced Universal SYBR Green Supermix (Biorad) and a CFX96 Real-Time PCR Detection System (Biorad). Thermocycler parameters were 95 °C for 30 s followed by 40 cycles of 95 °C for 10 s, 60 °C for 30 s with plate reading, and a 65–95 °C melt curve in 5 s increments. Gene expression was calculated by standard curve method using target gene expression relative to 18S mRNA. PCR primer sequences are listed in Supplemental Table S1.

### DNA sequencing

DNA was extracted from VM–EC (CD31^+^) and non-endothelial cell population (CD31^−^) using QIAamp DNA Mini Kit (Qiagen). Genomic DNA from peripheral blood, plasma, serum, or buccal swab was extracted using Purelink Genomic DNA Mini Kit (Invitrogen). DNA quality and quantity were determined using a Nanodrop 2000c Spectrophotometer. 200 ng of DNA was used for PCR using GoTaq Polymerase Master Mix (Promega). *TIE2* (*TEK*) coding sequence next-generation sequencing (NGS) and analysis was performed at the CCHMC DNA Sequencing and Genotyping Core following GeneRead Panel (Qiagen) sequence amplification.

To confirm *TIE2* mutations and identify *PIK3CA* mutations, DNA Sanger sequencing was performed as follows. Primers were used to amplify *TIE2* exon 17 and *PIK3CA* exons 7, 9, and 20 (Integrated DNA Technologies) (Supplemental Table S1). Amplification product was purified using QIAquick Gel Extraction Kit (Qiagen) and sequenced at CCHMC DNA Sequencing and Genotyping Core. Electropherogram peak results were visualized using CodonCode Aligner (CodonCode Corporation).

### Single cell-derived clone mutation analysis

VMK EC clones were obtained by diluting 1 cell/400 µl of EGM-2 20%FBS, 1% PSG medium, and 100 µL were seeded into each fibronectin-coated well of 96-well plates. Single colonies were then isolated and propagated until efficient cell numbers were met for DNA extraction, PCR amplification, and Sanger sequencing for mutation analysis.

### Xenograft model for VM

After cell expansion, 2.5–3.5 × 10^6^ VM–EC, HUVEC, HUVEC–TIE2–L914F, and HUVEC–TIE2–WT were suspended in Matrigel™ (Corning) and injected subcutaneously on both flanks of 6- to 7-week-old male athymic nu/nu mice (Envigo). Lesions were dissected after 9 days, fixed in 10% formalin, and paraffin embedded. After Hematoxylin and Eosin (H&E) staining, five images were taken randomly per section using EVOS (Life Technologies), followed by vascular density (vessels/mm^2^) and vascular area (%) quantification with ImageJ software.

### Immunostaining

Paraffin tissue sections were stained following antigen retrieval with citrate buffer, pH 6.0, and blocking using 5% horse serum in PBS. Immunohistochemistry was performed with biotin-conjugated Ulex europaeus I (UEA)(1:100, Vector Laboratories). Peroxidase was quenched using 3% H_2_O_2_(Sigma) prior incubation with streptavidin-conjugated horseradish peroxidase (HRP) (1:200, Vector Laboratories) followed by diaminobenzidine (DAB) (Vector Laboratories). Samples were mounted with VectaMount (Vector Laboratories). Immunofluorescence was performed using UEA, Griffonia simplicifolia Isolectin B4 (IB4) (1:50 and 1:100, Vector Laboratories), and anti-αSMA (1:500, Sigma). FITC/ Texas Red-conjugated secondary antibodies (1:200, Vector Laboratories) were used. Samples were mounted using Prolong Gold with DAPI. Images were acquired using Nikon C2 confocal microscope and NIS-Elements C imaging (Nikon).

### Immunoblotting

VM–EC and HUVEC were starved for 3 h in Endothelial Basal Medium-2 (EBM-2) supplemented with 1% FBS and then treated with Angiopoietin 1 (ANGPT1) 1 µg/ml (R&D Systems) for 15 min. Cells were then washed with PBS then lysed using RIPA buffer (Boston Bioproduct) with phosphatase inhibitor cocktail (Roche). Lysates were subjected to SDS-PAGE and transferred to Immobilon-P membrane. Membranes were probed with antibodies against the following: phospho-TIE2(Y992), TIE2, phospho-AKT (Ser473), AKT, phospho-ERK 1/2, ERK 1/2 (all 1:1000 Cell Signaling Technologies), and β-actin (1:2000, Sigma Aldrich). Membranes were incubated with peroxidase-conjugated secondary antibodies (1:5000, Vector Laboratories). Antigen–antibody complexes were visualized using ECL (Pierce) and chemiluminescent sensitive film. Band intensity was analyzed with ImageJ software.

### Statistical analysis

Results are reported as means ± standard deviation (SD). Unpaired student *t* test was performed, and *p* values reported with differences between groups considered significant at *p* ≤ 0.05.

## Electronic supplementary material

Below is the link to the electronic supplementary material.

Supplementary material 1 (PDF 78861 KB)

## Abbreviations

VM: Venous malformation
EC: Endothelial cells
PI3K: Phosphoinositide 3-kinase
