# Supplementary material for: A xenograft model for venous malformation
Source: Angiogenesis. 2018 May 21;21(4):725–35. doi: 10.1007/s10456-018-9624-7 (PMC6203618; doi:10.1007/s10456-018-9624-7)
Supplement: Supplementary file 1 — Supplementary material 1 (PDF 78861 KB) [file 10456_2018_9624_MOESM1_ESM.pdf]

Supplementary Material for

A Xenograft Model for Venous Malformation

Jillian Goines[1], Xian Li[1], Yuqi Cai[1], Paula Mobberley-Schuman[2], Megan Metcalf[2], Steven J. Fishman[3], Denise M. Adams[4], Adrienne M. Hammill[2,5], and Elisa Boscolo[1,5*]

[1] Division of Experimental Hematology and Cancer Biology, and [2] Division of Hematology, Cancer and Blood Diseases Institute, Cincinnati Children's Hospital Medical Center, Cincinnati, OH, USA

[3] Departments of Surgery, [4]Department of Hematology, and the Vascular Anomalies Center, Boston Children's Hospital, Harvard Medical School, Boston, MA, USA

[5] Department of Pediatrics, University of Cincinnati College of Medicine, Cincinnati, OH, USA

*Corresponding author: elisa.boscolo@cchmc.org

The PDF File includes:

Fig. S1. Morphology of cultured patient-derived EC at low and high passage.

Fig. S2. *TIE2* and *PIK3CA* sequencing of 7 VMK-EC clonal populations.

Fig. S3. Histology of patient Venous Malformation.

Fig. S4. Lesion explants from injected HUVEC-TIE2-L914F and control HUVEC-TIE2-WT (wild type).

Fig. S5. IB4 staining of VM xenograft lesion sections.

Fig. S6. Staining of control tissues.

Table S1. List of Primers for DNA Sanger sequencing.

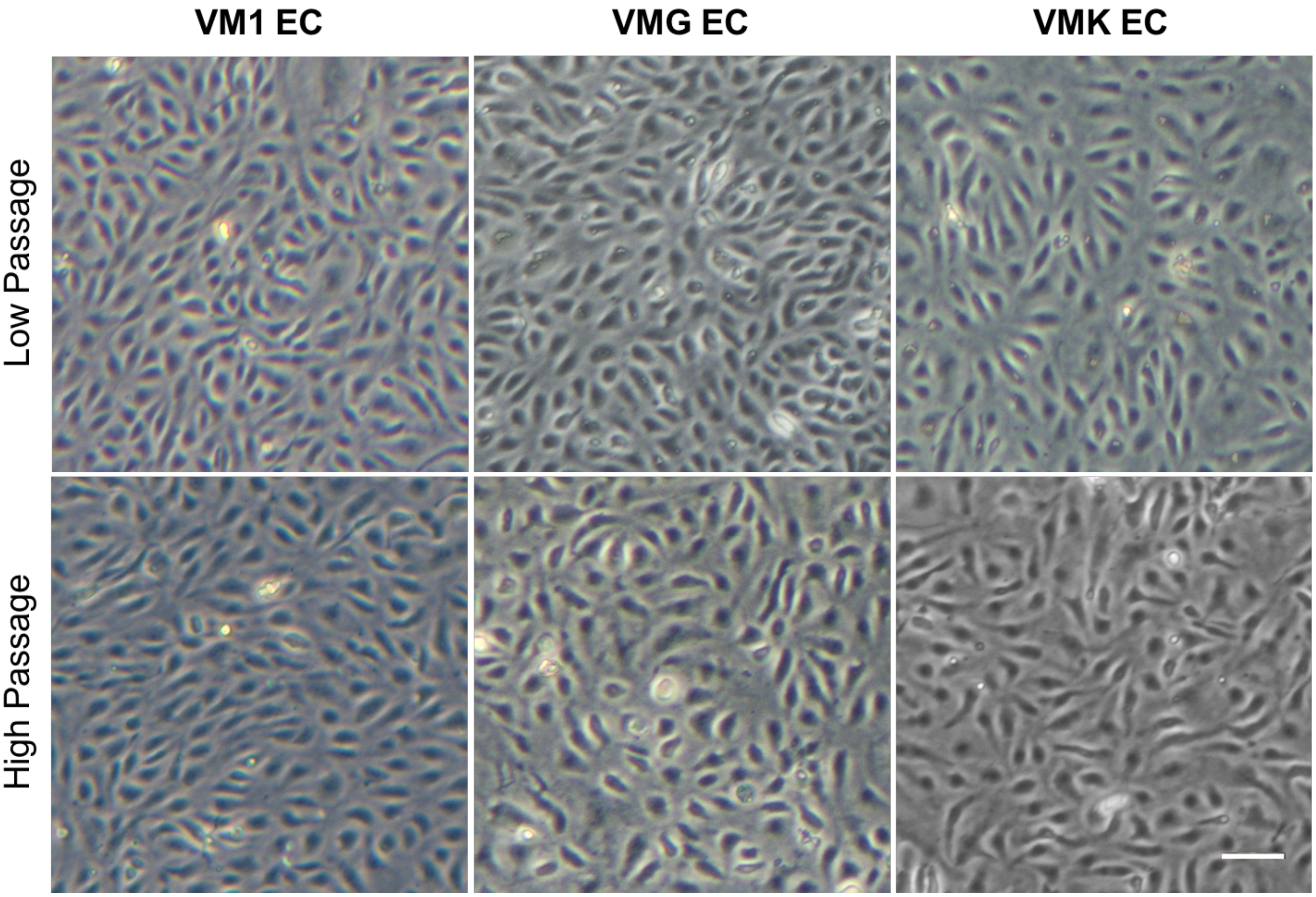

Supplemental Fig.S1. Morphology of cultured patient-derived EC at low and high passage. Morphological analysis of Venous Malformation endothelial cells (VM-EC) from different patients (VM1, VMG, VMK) at low passage (p3) and high passage (p7).

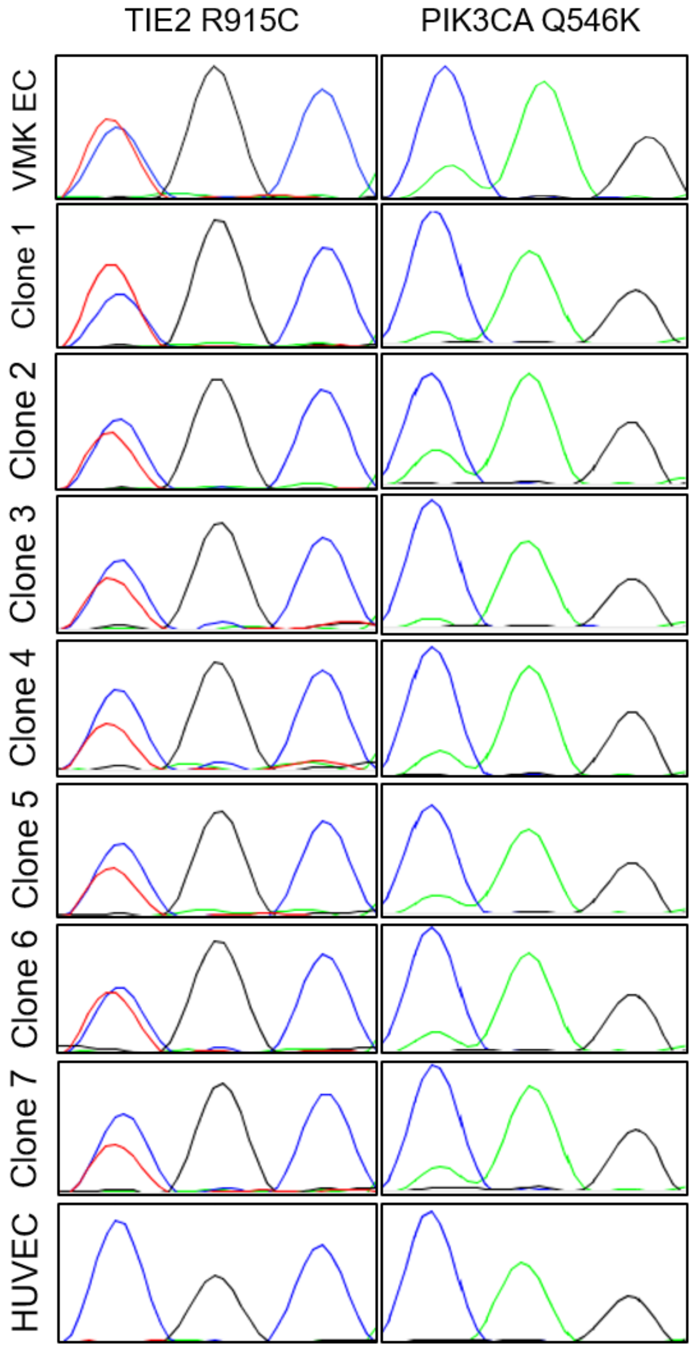

Supplemental Fig. S2. *TIE2* and *PIK3CA* sequencing of 7 VMK EC clonal populations.
DNA Sanger sequencing of VMK EC clone derived from 1 single cell. Presence of a double peak at *TIE2* c.2743C>T (p.R915C) (left) and c.1636C>A (p.Q546K) (right) in VMK EC population and in 7/7clonal populations. HUVEC did not show peaks for these variants.

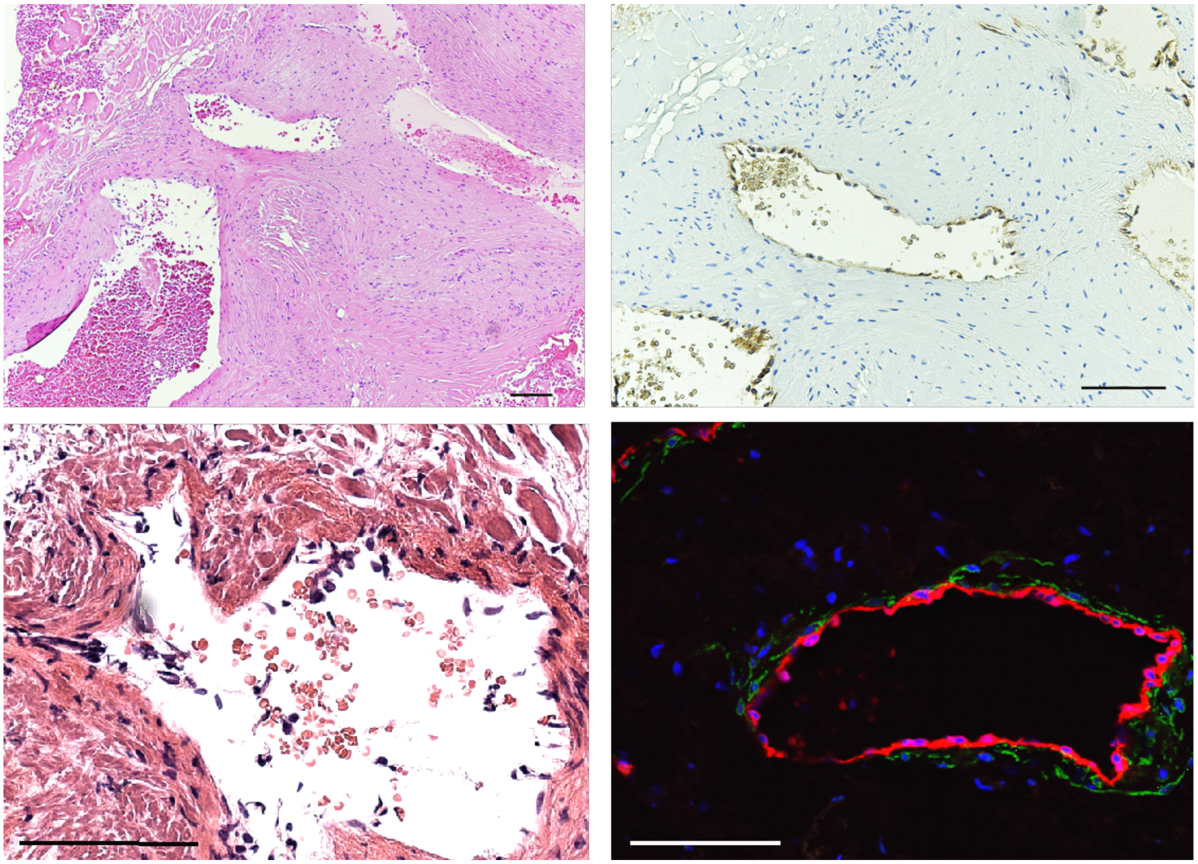

Supplemental Fig.S3. Histology of patient Venous Malformation.
Patient-derived Venous Malformation tissue subjected to hematoxylin and eosin staining (H&E) (left), immunohistochemical staining for Ulex europaeus Agglutinin I (UEA) (brown color) (top, right) and immunofluorescence staining for UEA (red) and smooth muscle alpha-actin (αSMA) (green) (bottom, right). Scale bar 100μm.

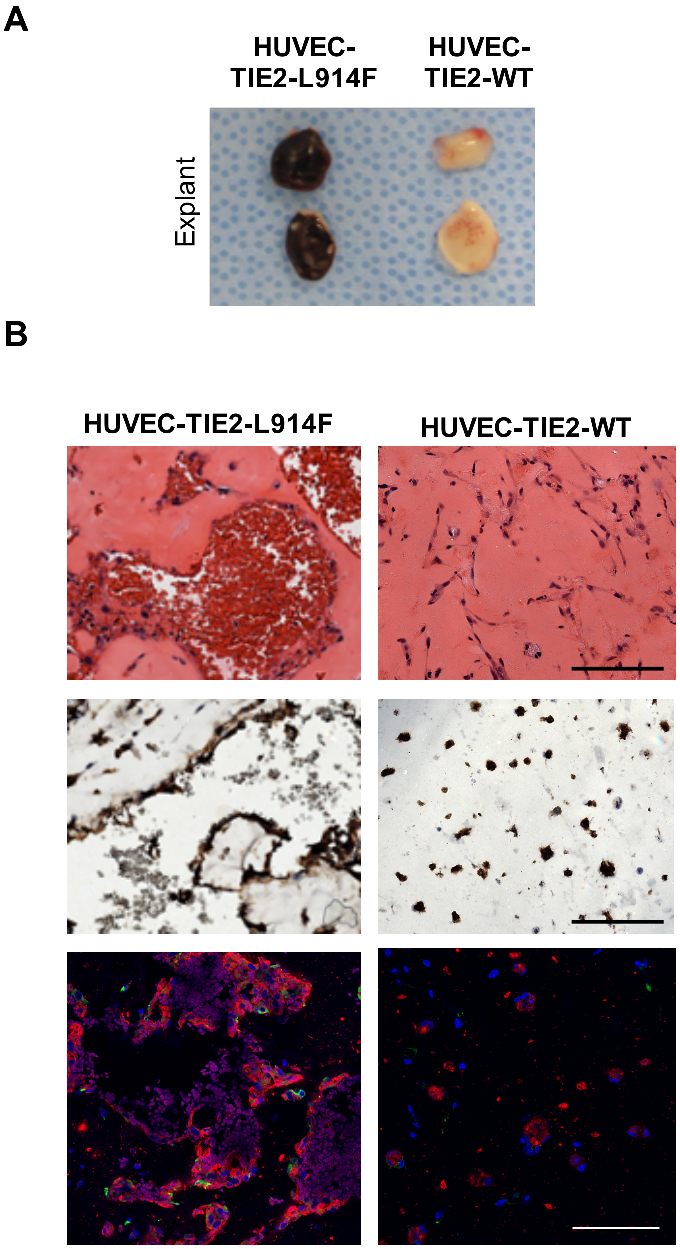

Supplemental Fig.S4. Lesion explants from injected HUVEC-TIE2-L914F and control HUVEC-TIE2-WT (wild type).

HUVEC-TIE2-L914F and HUVEC-TIE2-WT cells were injected subcutaneously on both backsides of immune-deficient mice. **A-** Lesion explant photo, **B-** staining of hematoxylin and eosin (H&E) (top), immunohistochemistry (IHC) of Ulex europaeus I (UEA) (middle), and immunofluorescence (IF) of UEA (red) and αSMA (green), nuclei (blue) (bottom); Scale bars: H&E**,** IHC and IF 100μm

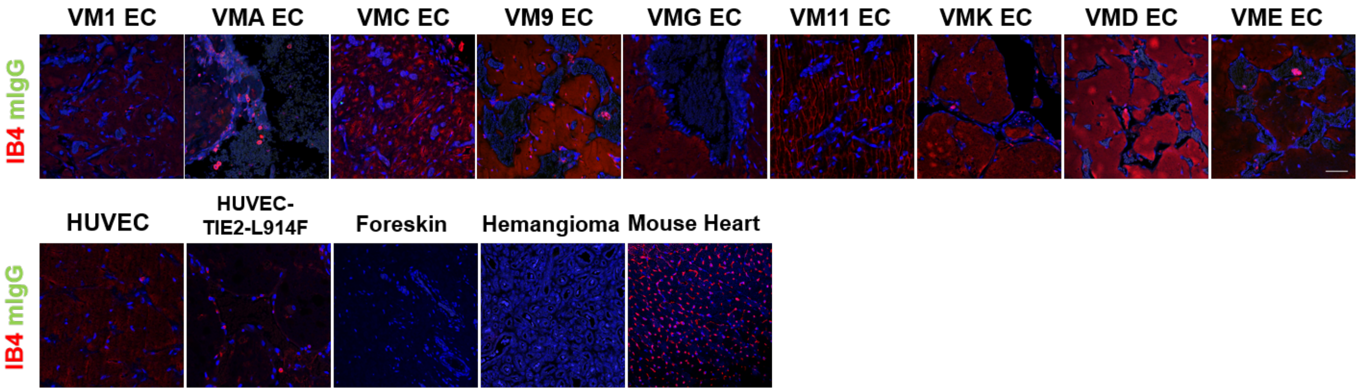

Supplemental Fig.S5. IB4 staining of VM xenograft lesion sections.
VM-EC xenograft explants subjected to immunofluorescence staining for Griffonia simplicifolia Isolectin B4 (IB4) (red), DAPI for nuclei (blue). Controls are HUVEC, HUVEC-TIE2-L914F explants, human foreskin, patient-derived infantile hemangioma and mouse heart (positive control for IB4). Scale bar 50μm.

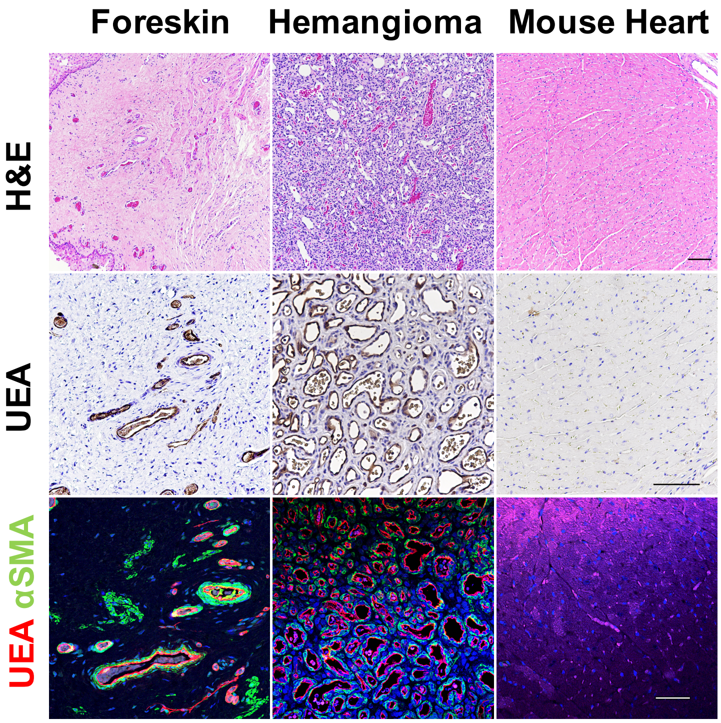

Supplemental Fig.S6. Staining of control tissues.
Human foreskin, patient-derived infantile hemangioma and mouse heart subjected to hematoxylin and eosin staining (H&E) (top), immunohistochemical (IHC) staining for Ulex europaeus Agglutinin I (UEA) (brown color) (center), and immunofluorescence (IF) staining for UEA (red) and smooth muscle alpha-actin (αSMA) (green) (bottom). Scale bar H&E, IHC 100μm; IF 50μm.

Supplemental Table S1. List of Primers for DNA Sanger sequencing.

Primer	Sequence
TIE2 Exon 17 mutations	F- 5' TGGTGTTGCTAGATGTGTTT<br>R- 5' TTTTGGCTCAAGTAGTCCAT
PIK3CA Exon 21 mutations	F- 5' ACATTCGAAAGACCCTAGCC<br>R- 5' ATGCTGTTCATGGATTGTGC
PIK3CA Exon 8 mutations [1]	F- 5' GGGGAAAAAGGAAAGAATGG<br>R- 5' TGCTGAACCAGTCAAACTCC<br>Seq- 5' TGAATTTTCCTTTTGGGGAAG
PIK3CA Exon 10 mutations	F- 5' GATTGGTTCTTTCCTGTCTCTG<br>R- 5' CCACAAATATCAATTTACAACCATTG<br>Seq- 5' TTGCTTTTTCTGTAAATCATCTGTG
CD31 (qRT-PCR)	F- 5' GACATGGCAACAAGGCTGTG<br>R- 5' CGGGCTTGGAAAATAGTTCTGT
VWF (qRT-PCR)	F- 5' CCGATGCAGCCTTTTCGGA<br>R- 5' TCCCCAAGATACACGGAGAGG
VE-Cadherin (qRT-PCR)	F- 5' GTTCACGCATCGGTTGTTCAA<br>R- 5' CGCTTCCACCACGATCTCATA
18S (qRT-PCR)	F- 5' GTCTGTGATGCCCTTAGATG<br>R- 5' AGCTTATGACCCGCACTTAC

BIBLIOGRAPHY

1. Samuels, Y., et al., *High frequency of mutations of the PIK3CA gene in human cancers.* Science, 2004. **304**(5670): p. 554.
